# Bacterial Infection Increases Reproductive Investment in Burying Beetles

**DOI:** 10.3390/insects6040926

**Published:** 2015-10-30

**Authors:** Catherine E. Reavey, Farley W. S. Silva, Sheena C. Cotter

**Affiliations:** 1 School of Biological Sciences, Queen’s University Belfast, MBC, 97 Lisburn Road, Belfast BT9 7BL, UK; E-Mails: creavey01@qub.ac.uk (C.E.R.); farleyw@gmail.com (F.W.S.S.); 2 Lancaster Environment Centre, Lancaster University, Lancaster LA1 4YQ, UK; 3 Department of Entomology, Universidade Federal de Viçosa, Viçosa 36570-900, Brazil; 4 School of Life Sciences, University of Lincoln, Brayford Pool, Lincoln LN6 7TS, UK

**Keywords:** bacteria, ecological immunology, insect, lysozyme, *Nicrophorus*, parental care, phenoloxidase, reproduction, survival, tolerance

## Abstract

The *Nicrophorus* genus lives and breeds in a microbe rich environment. As such, it would be expected that strategies should be in place to counter potentially negative effects of the microbes common to this environment. In this study, we show the response of *Nicrophorus vespilloides* to the common soil bacterium, *Bacillus subtilis*. Phenoloxidase (PO) levels are not upregulated in response to the challenge and the bacteria are observed to multiply within the haemolymph of the host. Despite the growth of *B. subtilis*, survival is not affected, either in virgin or in breeding beetles. Some limit on bacterial growth in the haemolymph does seem to be occurring, suggesting mechanisms of resistance, in addition to tolerance mechanisms. Despite limited detrimental effects on the individual, the challenge by *Bacillus subtilis* appears to act as a cue to increase reproductive investment. The challenge may indicate a suite of negative environmental conditions that could compromise future breeding opportunities. This could act as a cue to increase parental investment in the current bout.

## 1. Introduction

Trade-offs between life-history traits are widely studied in evolutionary biology. Costs of reproduction are well documented [1,2,3] and recently attention has focused on ecological immunology, providing evidence for the costliness of immune function. These costs may be evolutionary costs [4,5], maintenance costs [6], deployment costs [7,8] or autoimmune costs [9]. As investment into both reproduction and immunity are costly, they often trade-off with each other [10,11,12]. Immune investment has only recently been integrated into a life-history framework; therefore, studies on trade-offs between reproduction and immune function are important.

Insects provide us with excellent model systems to test evolutionary questions, and here they will be used to elucidate the reproduction-immunity trade-off, using a host-bacteria interaction that is likely to occur in the wild. While their immune system is “simpler” than that of vertebrates, many mechanisms are conserved, allowing results to be extrapolated to vertebrates [13].

In insects, the immune response consists of two main components: cellular immunity and humoral immunity. The cellular response is largely constitutive; there is a standing level of haemocytes present. These act in a generalised manner, via the mechanisms of phagocytosis of microparasites, nodulation of clumps of microparasites and encapsulation of macroparasites [14], and are the first defence present upon invasion. Activation of the prophenoloxidase (pro-PO) cascade [14] is central to the constitutive response, resulting in an upregulation of PO [14]. This leads to the production of melanin [15], a substance used in the encapsulation response. Phenoloxidase (PO) also plays a key role in non-self recognition [16], the coordination of the cellular response [14] and cuticular hardening [17]. The humoral arm is usually induced in response to infection and is more specific [18,19]. It includes lysozyme and other smaller antimicrobial peptides (AMPs) [20]. While the PO cascade is predominantly associated with cell-based immunity, it also contributes to humoral immune function with both the intermediate products of the cascade (quinones) [21] and melanin having antimicrobial activity in the haemolymph [22,23].

Virulence, the degree to which a microorganism can harm the host, is important when studying the effect of live immune challenges. Some very virulent entomopathogens have co-evolved with insects and target their hosts with often species-specific strategies. Other non-species-specific microbes can still be very virulent, despite not having the specialised invasion mechanisms of entomopathogens. In the arms race against pathogens/microorganisms both resistance (the ability to limit or clear a parasite load) and tolerance (the ability to limit the harm caused by a given parasite load) are important for the host [24].

In this study, we consider the effect of a live bacterial challenge on the personal immune proxy PO as well as potential life-history trade-offs between immunity, reproduction (including social immunity) and survival, in the burying beetle *Nicrophorus vespilloides*. Potential trade-offs between immune investment and reproductive investment and their fitness consequences are central to evolutionary theory, and studying a live bacterial challenge, as opposed to an immune elicitor, allows us to make better inferences about real ecological scenarios.

**Figure 1:**
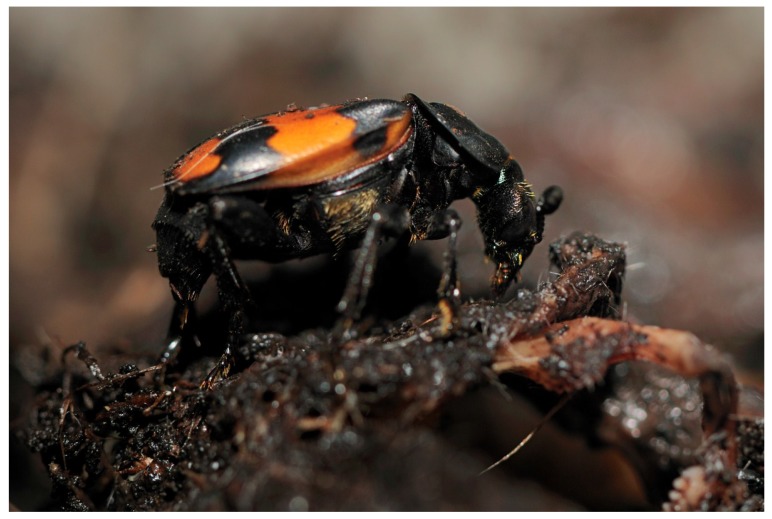
*Nicrophorus vespilloides* preparing the carcass. Photo courtesy of O. Kruger.

Burying beetles (Figure 1) are unusual in the insect class in that they exhibit extended biparental care. Following carcass location, the parents cooperate to bury the carcass underground and prepare it for consumption by their offspring by removing hair or feathers, shaping it into a ball and making an entry point for the larvae [25,26]. Many microorganisms will attempt to colonise the carcass, making *N. vespilloides* an interesting study organism for considering strategies by which to inhabit a microbe rich environment. The beetles coat the carcass with antimicrobial anal exudates to delay decomposition [27]. This is a form of social immunity [28] and improves larval survival [29]. It has also been shown to be costly [30]. Eggs are laid in the surrounding soil [26]. Two to three days after egg laying in *N. vespilloides*, the larvae crawl to the carcass, are fed predigested food from their parents and are protected from predators and competitors [26,27,28,29,30,31]. The larvae disperse after roughly six days, and pupation begins, lasting around 20 days [26,27,28,29,30,31]. Parental care improves offspring growth and survival in *N. vespilloides* [32], so we can therefore use number and mass of larvae produced as a proxy for parental investment.

While the body of evidence for social and personal immunity is growing in this genus [27,28,29,30,33,34,35,36,37,38,39], no studies using a live challenge and considering any effects on life history have been documented to date. Although studies using immune elicitors are essential when considering deployment costs, overall costs may be very different when using actively replicating microorganisms, which can cause direct physical damage to the host as well as producing a suite of harmful toxins. Both resistance and tolerance strategies are invariably costly processes [40,41]. If a strategy to combat the invader did not occur, aside from the detrimental effects of toxins *etc.*, eventually the microorganism would take up all the physical space in the haemocoel and would co-opt host resources, so some level of action must be employed. Variation in both tolerance and resistance can be often observed at both the level of the species and the individual.

Resistance may arise through behavioural traits, e.g., avoidance of the parasites, physical traits, e.g., the exoskeleton in insects, or internal immune or physiological factors, e.g., a powerful immune response or specific gene-gene interactions between parasite and host involving recognition, which may result in resistance without having to further employ immune function [42]. Variation in tolerance, both across and within taxa can be driven by different mechanisms, such as nutrition, efficient tissue repair, the ability to detoxify by-products of bacterial replication and the use of commensal/mutualistic bacteria [43,44].

Here, this area is addressed by challenging *N. vespilloides* with a bacterial infection of *Bacillus subtilis*. *B. subtilis* was selected for the direct challenge based on a study by Hall *et al.* [45], showing that oral and anal secretions in this species inhibit *B. subtilis*. Although social and personal immune function are under different selective pressures and are likely to vary accordingly, this seems sufficient reasoning to use *B. subtilis* as a personal immune challenge as it would be ecologically relevant. Furthermore, we previously found a personal immune response to an elicitor challenge containing peptidoglycan (PEP) from *B. subtilis* [39], so it seems a good candidate for challenging the immune system.

As burying beetles have evolved to live in a microbe rich environment, they may employ unique strategies resulting in resistance or tolerance to bacterial challenge. Presence/absence of bacteria may also be a central cue to their behaviours. For example, a study by Cotter *et al.* [34] used heat-killed bacteria, *Micrococcus luteus*, as a challenge prior to breeding. This seemed to signal to the beetle that “risk of death” was higher, resulting in a lifting of reproductive restraint and increased larval output, in both young and old beetles. However, we do not know if the same effect would be seen when the beetles have to pay the costs associated with hosting an actively replicating bacteria.

These experiments aim to build on previous work on immune elicitors (LPS, PEP and heat-killed bacteria) and wounding challenges [34,38,39], to determine how a live bacterial challenge by injection of *B. subtilis* affects immune parameters, reproductive investment and survival, and ultimately add to evolutionary theory regarding how the life-history traits of reproduction and immune investment are balanced. We hypothesise that immune function will be upregulated upon live bacterial challenge. We might expect a decline in reproductive output if bacterial replication utilises host resources and if host resources are also diverted from parental investment to immune function, though this may be counteracted by a lifting of reproductive restraint. Furthermore, it is likely that longevity will be affected by the challenge, either through the costs of dealing with an infection, or direct bacteria-induced mortality.

## 2. Experimental Section

### 2.1. Nicrophorus vespilloides

The colony was founded in February 2011 from an outbred colony maintained in the Zoology department at the University of Cambridge. The colony was housed at 20 °C under a 16:8 light:dark cycle, and fed twice weekly *ad libitum* with minced beef. Non-breeding adult beetles were maintained in individual boxes containing moist compost (measuring 12 × 8 × 2 cm) and during breeding, each pair was placed together in a breeding container (17 × 12 × 6 cm), 1/3 filled with moist compost and provided with a newly defrosted mouse carcass. When breeding, the beetles were placed in a cupboard to simulate underground conditions.

Larvae were removed from the breeding container immediately upon dispersal from the carcass, typically 8–10 days after the parents were paired. They were weighed, counted and placed individually in compartments of 25 cell petri dishes, with different petri dishes used for each family. The containers were topped up with moist compost and the larvae left to pupate. Eclosion occurs around 20 days following dispersal, after which the beetles were set up in their individual containers and were either used as colony beetles or used in later experiments.

### 2.2. Experiment 1: Measuring the Immune Response to Bacterial Challenge

#### 2.2.1. Do Phenoloxidase (PO) Levels Change in Response to Bacterial Challenge?

Non-breeding, virgin, three-week old beetles of both sexes (1:1 M:F) were assigned to one of five treatment groups: (1) Handled; (2) Injected with 1 µL of autoclaved insect ringer’s solution (wounded); (3) Injected with 1 µL of autoclaved nutrient broth; (4) Injected with 1 µL of heat-killed *B. subtilis* (1.6 × 10^6^ cells/mL) in nutrient broth and (5) Injected with 1 µL of *B. subtilis* (1.6 × 10^6^ cells/mL) in nutrient broth. The treatments were applied by injection with a Hamilton syringe into the cuticle behind the pronotum. While the insect ringer’s solution and nutrient broth were autoclaved, an open wound may be vulnerable to bacterial entry subsequent to treatment application, especially as soil is a microbe rich environment. Twenty-four hours after immune challenge, haemolymph samples were obtained from each beetle and processed as stated below. Thirty-seven beetles were used per treatment groups and 179 samples obtained in total from a possible 185. The samples were obtained in two blocks staggered two days apart for logistical reasons.

#### 2.2.2. How Do Bacterial Levels Change in the Haemolymph over Time?

Two-week old, non-breeding beetles of both sexes (1:1 M:F) were assigned to one of five groups, with 10 beetles per group. All beetles were injected with 2 µL of *B. subtilis* in nutrient broth (8 × 10^5^ cells/mL) on the cuticle behind the pronotum, using a Hamilton syringe. Haemolymph was then collected from all beetles in a group at either: 1, 6, 12, 24 or 72 h post infection in discrete groups of beetles. Following collection, the haemolymph samples were plated on 1.5% nutrient agar plates and the number of bacterial colony forming units quantified as described below.

#### 2.2.3. Does Bacterial Challenge Affect Survival in Virgin Beetles?

Five treatment groups were established: (1) Handled; (2) Injected with 1 µL of autoclaved nutrient broth; (3) Injected with 1 µL of *B. subtilis* (1.6 × 10^5^ cells/mL) in nutrient broth; (4) Injected with 1 µL of *B. subtilis* (8 × 10^5^ cells/mL) in nutrient broth and (5) Injected with 1 µL of *B. subtilis* (1.6 × 10^6^ cells/mL) in nutrient broth. The treatments were applied to two-week old virgin beetles, both male and female (1:1 M:F). The beetles were fed twice weekly until death and survival recorded. Thirty beetles were assigned to each treatment group. Three beetles escaped mid-experiment, resulting in survival data for 147 beetles.

### 2.3. Experiment 2: Does Bacterial Challenge Affect Reproductive Investment and Subsequent Survival?

Two-week old female burying beetles were paired with an unrelated male and placed in a breeding container in the presence of a mouse. The male was removed on day 2 of the breeding bout to prevent assistance in parental investment. The females were assigned to one of three groups: (1) Injected with autoclaved nutrient broth; (2) Injected with 1 µL of *B. subtilis* (8 × 10^5^ cells/mL) in nutrient broth and (3) Injected with 1 µL of *B. subtilis* (1.6 × 10^6^ cells/mL) in nutrient broth. Different treatment groups were used compared to Experiment 1 as we wanted to determine if there was a dose effect of bacteria on reproduction, rather than considering effects of wounding or immune elicitor treatments. These treatments were applied on day 2 of the breeding bout. On day 4 of the breeding bout, 48 h after treatment application, exudate samples were obtained from the females, and processed to determine any potential changes in lytic activity in response to bacterial challenge. Dispersing larvae were weighed and counted to determine reproductive output and so parental investment. The females were then fed twice weekly until death and subsequent survival recorded (each female was only bred once during their lifespan). Seventy-five beetles were used across the three treatment groups. Ten beetles did not breed and were omitted from the reproductive analysis. One beetle escaped prior to death and so was omitted from the survival analysis. Exudate samples were not obtained for all breeding beetles. Fifty-seven exudate samples were obtained from the 65 pairs that bred and these individuals were used in the analysis.

### 2.4. Haemolymph Sampling

Haemolymph was obtained from *N. vespilloides* by piercing the cuticle behind the pronotum with a sterile needle and the haemolymph collected with a pipette as it was released. The haemolymph was diluted in a 1:1 ratio with anticoagulant buffer (EDTA anticoagulant in PBS-pH 7.4) and stored in a freezer (−20 °C) until analysis.

### 2.5. Phenoloxidase (PO) Assay

Following defrosting of the haemolymph samples, 2 µL of haemolymph/anticoagulant buffer solution was added to 500 µL of PBS (pH 7.4), 100 µL of this solution was placed in a well of a 96-well microplate with 100 µL of 10 mM dopamine as a substrate. Readings were taken every 10 s for 3 min at 490 nm and 25 °C on a Thermo Scientific Multiscan Spectrum spectrophotometer. This range accounted for the linear stage of the reaction [46]. The maximum rate of reaction was then used as an approximation of PO level.

### 2.6. Exudate Sampling

A capillary tube was used to catch the exudates produced from most beetles when disturbed or handled. Tapping the abdomen of the beetle was usually sufficient to obtain an adequate sample. The sample was then blown into an Eppendorf and stored prior to processing at −20 °C.

### 2.7. Lytic Assay

Bacterial agar plates, consisting of 10 mL of 1.5% agar:potassium phosphate buffer (2:1) and 50 mg of freeze-dried *Micrococcus luteus*, were used to determine lytic activity. Following defrosting and vortexing of the stored exudate, 2 mm diameter holes were punched in each plate and each well filled with 1 µL of exudate, with two technical replicates per sample. The plates were incubated at 33 °C for 24 h, and the clear zones around the samples were measured using digital callipers. Lytic activity (mg/mL) was then calculated from a serial dilution of a hen egg white lysozyme standard.

### 2.8. Bacterial Standardisation and Quantification

Viability of bacteria for initial injection was ensured by quantification of log phase *B. subtilis*. The bacteria were quantified using a haemocytometer. Following collection of haemolymph samples, 2 µL of haemolymph was taken and diluted in 50 µL of PBS buffer. Then, 25 µL of the resulting solution was spread on a nutrient agar plate, with two replicates per sample (beetle). The plates were incubated at 33 °C for 24 h, after which the number of colony forming units per plate was counted.

### 2.9. Statistical Analyses

For all but the survival data, we used general linear mixed models, with log transformations when inspection of residuals suggested heteroscedasticity or deviations from normal distribution. Starting from models including all possible terms, we applied model selection by comparing nested models with ANOVA. Survival data for Experiment 1c and 2 were analysed using survival analysis with a Weibull distribution. GLMMs were used in Experiment 1a, 1b and 2. PO values were log transformed in the analysis. In Experiment 1a considering PO activity, the model included treatment group as a factor with five levels, sex as a factor and the sex*treatment interaction. Block was also included as a factor and the block-treatment interaction. In Experiment 1b considering bacterial growth, time was included in the model as a continuous variable. In Experiment 1c, a survival analysis was carried out including the terms for challenge (five-levelled factor) and sex as a factor. The bacterial challenge in Experiment 2 was included as a factor with three levels, carcass weight as a continuous variable and the interaction between the bacterial treatment and carcass weight. In various models, the treatment groups were pooled, as discussed in the results section. Three reproductive proxies (brood weight, mean larval weight and total larval number) as well as lytic activity were considered. A survival analysis included treatment (three-levelled factor), reproductive output (continuous variable) and carcass weight (continuous variable) in the model. All analyses were carried out using R 3.1.2 (Development Core Team, R Foundation for Statistical Computing, Vienna, Austria, 2013).

## 3. Results

### 3.1. Experiment 1: Measuring the Immune Response to Bacterial Challenge

#### 3.1.1. Do Phenoloxidase (PO) Levels Change in Response to Bacterial Challenge?

**Figure 2:**
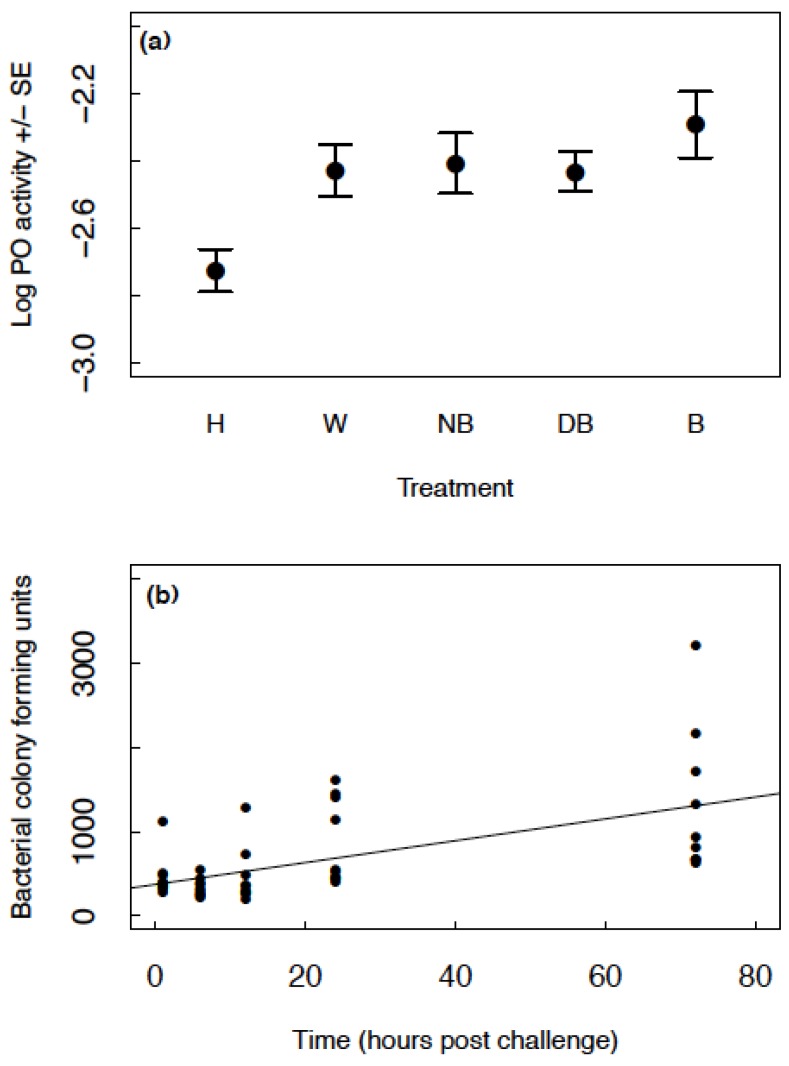
(**a**) The mean raw data for log PO ± SE across the different treatment groups; handled (H), injected with insect ringer’s solution (W), injected with nutrient broth (NB), injected with heat-killed *B. subtilis* (DB) and injected with live *B. subtilis* (B); (**b**) The bacterial colony forming units produced from haemolymph samples at time points 1–72 h post infection. The black points show the raw data and the line illustrates the fitted values of the model.

PO levels increased in all groups (wounded, injected with nutrient broth, injected with dead bacteria and injected with bacteria) relative to the handled control group (GLMM: handled estimate = −2.726, other group estimates >−2.445, *t* < 4.270, *p* < 0.008; Figure 2a). There was no difference in PO levels between the wounded, injected with nutrient broth, injected with dead bacteria and injected with live bacteria groups (Figure 2a). There was no effect of sex on PO (GLMM: F_1,149_ = 0.40, *p* = 0.528) and no interaction between sex and treatment group (GLMM: F_4,163_ = 0.36, *p* = 0.838). There was no difference between blocks (GLMM: F_1,43_ = 1.77, *p* = 0.190) and no interaction between block and treatment group (GLMM: F_4,140_ = 0.87, *p* = 0.485).

#### 3.1.2. How Do Bacterial Levels Change in the Haemolymph over Time?

Colony forming units increased with time in a linear manner (GLMM: estimate = 13.310, SE = 2.695, *t*_41_ = 4.938, *p* < 0.001; Figure 2b). Figure 2b shows CFUs produced from approximately 1 µL of haemolymph. If we (conservatively) estimate around 20 µL of haemolymph in the haemocoel, these values would range from approximately 6000–60,000 bacterial cells present in the haemocoel across time. The initial injection of 2 µL of 8 × 10^5^ cells/mL is equivalent to an injection of 1600 cells. Therefore, even at one-hour post challenge, there was a significant increase in bacterial load.

#### 3.1.3. Does Bacterial Challenge Affect Survival in Virgin Beetles?

There was no effect of injection of nutrient broth or injection of the different bacterial solutions on the survival of virgin beetles relative to handled controls (*χ*^2^_[4]_ = 7.35, *p* = 0.120). Sex did not affect survival (*χ*^2^_[2]_ = 3.07, *p* = 0.215). Beetles in the handled, injected with nutrient broth, injected with the low, moderate or high bacterial load groups lived on average for 63.7 ± 3.3 (SE), 59.5 ± 3.0, 64.1 ± 2.2, 57.9 ± 2.5, 59.1 ± 2.3 days post eclosion, respectively.

### 3.2. Experiment 2: Does Bacterial Challenge Affect Reproductive Investment and Subsequent Survival?

**Figure 3:**
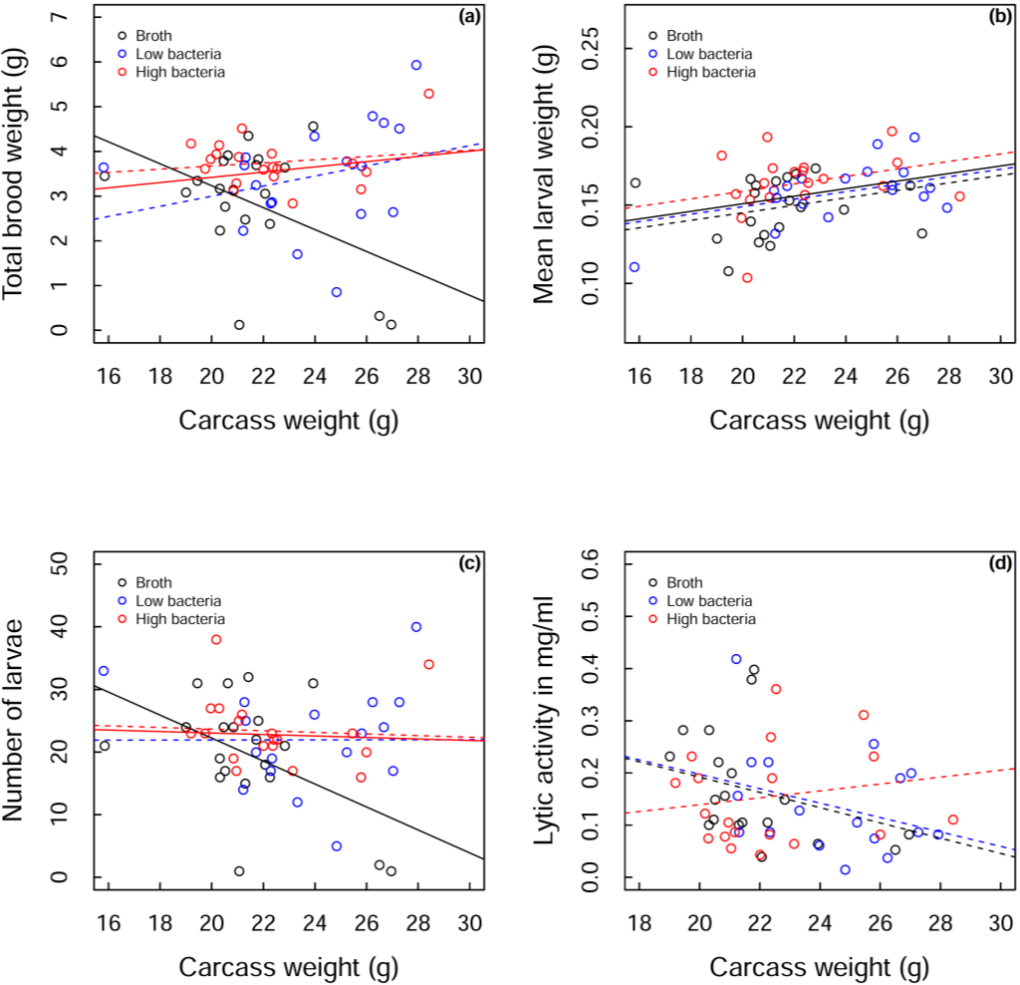
Measurements of three reproductive outputs, (**a**) total brood weight; (**b**) mean larval weight and (**c**) number of larvae produced; as well as (**d**) lytic activity against carcass weight for each of the three treatment groups: broth (black), low bacteria (blue) and high bacteria (red). Raw data is shown in open circles. Dotted lines show fitted values for each treatment group. The fitted values for the pooled bacterial treatment group are shown in solid red lines against solid black lines for the nutrient broth control group. A solid black line also shows the overall fitted values of the model in (**b)**.

Beetles that did not reproduce were omitted from the analysis (nutrient broth, *n* = 4, low bacteria, *n* = 4, high bacteria, *n* = 2). Initial visual inspection of the data suggested that the measures of reproductive output (total brood weight, mean larval weight and number of larvae, see Figure 3a–c, dotted lines) were very similar for the two bacterial treatment groups. For the analyses, these groups were therefore pooled as a “bacteria treatment”. The total weight of larvae produced by the different treatment groups depended on the weight of the carcass; a greater total weight of larvae was produced at the higher carcass weights for the bacteria-treated beetles compared to those injected with nutrient broth. This was mostly driven by a sharp decline in total brood weight in control beetles as carcass size increased (GLMM: bacteria group estimate = 0.058, control estimate = −0.245, SE = 0.114, *t*_53_ = −2.657, *p* = 0.010; Figure 3a).

So is this change in total brood weight with increasing carcass size driven by control beetles producing fewer larvae on large carcasses, or producing smaller larvae, or a combination of the two? Mean larval weight increased with carcass size for both treatment groups (GLMM: estimate = 0.002, SE = 0.001, *t*_54_ = 2.706, *p* = 0.009; Figure 3b). There was no interaction between treatment group and carcass weight (GLMM: F_1,47_ = 0.63, *p* = 0.431; Figure 3b) and a marginal effect of treatment group (GLMM: F_1,43_ = 3.63, *p* = 0.063; Figure 3b). Similar to total brood weight, the number of larvae produced depended on the weight of the carcass. While the bacteria group maintained the number of larvae produced as carcass size increased, controls showed a reduction in the number of larvae with increasing carcass size (GLMM: bacteria group estimate = −0.119, control estimate = −1.834, SE = 0.839, *t*_53_ = −2.045, *p* = 0.046; Figure 3c), suggesting that the change in total brood weight is mostly driven by the number of larvae produced, alongside a marginal increase in mean larval weight in bacteria-treated beetles.

While the interaction may be driven by two data points at high carcass weights for control beetles, these points cannot be excluded from the analysis as they are the only controls in that region. Instead we analysed the main cluster of points (19–23 g carcass weights) and found that bacteria-treated beetles have marginally greater total brood weights than control beetles (GLMM: bacteria group estimate = 3.575, control estimate = 3.063, SE = 0.257, *t*_34_ = −1.996, *p* = 0.054) and that high bacteria-treated beetles have a significantly greater total brood weight than nutrient broth-treated beetles (GLMM: F_2,33_ = 3.71, *p* = 0.035), strengthening the case for injection of bacteria resulting in a lifting of reproductive restraint. Therefore, regardless of the carcass size, total brood weight increases with bacterial treatment. If we consider the full range of carcass weights, this pattern seems to be predominantly driven by the number of larvae being maintained on high carcass weights in bacteria-treated beetles, although there is a marginal increase in mean larval weight with bacteria-treated beetles. However, if we only consider carcass weights from 19–23 g, the pattern seems to be driven by a marginal increase in mean larval weight in bacteria-treated beetles (GLMM: bacteria group estimate = 0.159, control estimate = 0.148, SE = 0.006, *t*_30_ = −1.829, *p* = 0.077), rather than a change in larval number (GLMM: bacteria group estimate = 22.850, control estimate = 21.000, SE = 2.139, *t*_34_ = −0.865, *p* = 0.393). Despite the subtleties of the pattern across carcass weights, we can clearly observe a lifting of reproductive restraint following bacterial challenge.

Lytic activity was maintained with carcass size for the high bacteria treatment group, but there was a marginal decrease in both the low bacteria and the control groups relative to high bacteria group (GLMM: high bacteria group estimate = 0.007, low bacteria estimate = −0.014, SE = 0.011, *t*_48_ = −1.804, *p* = 0.078; control estimate = −0.015, SE = 0.012, *t*_48_ = −1.787, *p* = 0.081; Figure 3d).

There was no effect of treatment (*χ*^2^_[2]_ = 2.50, *p* = 0.290), carcass weight (*χ*^2^_[1]_ = 0.68, *p* = 0.410) or reproductive output (number of larvae: *χ*^2^_[1]_ = 0.35, *p* = 0.560; total weight of larvae: *χ*^2^_[1]_ = 2.41, *p* = 0.120; mean weight of larvae: *χ*^2^_[1]_ = 2.49, *p* = 0.110) on survival. Beetles injected with nutrient broth, low or high bacterial load lived on average for 64.8 ± 2.5 (SE), 64.4 ± 2.7 or 69.9 ± 2.2 days post eclosion, respectively.

## 4. Discussion and Conclusions

This study shows that although *N. vespilloides* is tolerant to *B. subtilis*, the presence of bacteria appears to act as a cue for increasing reproductive investment. In Experiment 1a, PO levels increased in all groups (wounded, injected with nutrient broth, injected with dead bacteria and injected with live bacteria) relative to the handled control group. This result clearly indicates that wounding increased PO levels, as demonstrated previously [39], but the presence of bacteria, either live or heat-killed, did not further increase PO levels. PO is involved in wound healing so the effects observed across the wounded, injected with nutrient broth, injected with dead bacteria and injected with bacteria are likely due to the wound healing process alone. As well as an involvement in wound healing, PO is likely upregulated prophylactically, as wounding could be a cue that infection is likely to occur.

While we expected PO to be upregulated further in response to a bacterial invasion, this was not the case. We know that the anal exudates from *N. vespilloides* prevent growth of *B. subtilis* [45], but this effect did not seem to extend to PO in this study. In all eco-immunological studies, it must be asked: what assays are appropriate for the research question of interest? While the biological relevance of an immune assay is sometimes assumed without investigation or any understanding of the immune response, PO is arguably one of the best overall indicators of insect immunity, due to its multifaceted role in immunity (as discussed earlier). While the mechanism of action of PO on microparasites such as bacteria is not clear [47], there is evidence that it plays a role in responding to bacteria; for example, one pathogenic bacterium, *Photorhabdus luminescens*, is known to produce a compound that inhibits PO production in *Manduca sexta* [48]. Therefore, the fact that at least some bacteria have evolved to overcome the action of PO gives some evidence that PO does seem to be an ecologically relevant immune parameter for bacterial studies. In *Daphnia magna*, higher PO resulted in a lower spore load of *P. ramosa* [49]. In mosquitoes, *Armigeres subalbatus*, the response to challenge by *M. luteus* consisted of melanisation, with PO appearing in many of the melanotic capsules enveloping bacteria [50]. However, in contrast, one study showed that a *Drosophila* mutant lacking a prophenoloxidase-activating enzyme was just as resistant to microbial infections as wild type flies [51]. A further understanding of the precise function of PO in insect defence would be very useful. Upregulation of PO may be beneficial in the case of this host-parasite interaction, but, perhaps for some reason, the organism “chooses” not to upregulate PO further. However, a previous study [39] showed a downregulation of PO upon injection with LPS and PEP, alongside an upregulation of the AMP defensin. Therefore, perhaps if defensin had been measured, or other AMPs, we would have observed an upregulation of immune function. Even when an organism can tolerate high levels of a microbe, some immune investment will be required; in this case, if no action was taken, *B. subtilis* would fill the haemocoel, but presumably phagocytosis/nodulation and AMPs limit the growth to some extent. Furthermore, the baseline levels of PO present are potentially having some effect on the bacteria. Further tests comparing growth of *B. subtilis* in artificial haemolymph to growth in the host haemolymph or growth in beetles that cannot produce PO would yield more information on whether the PO present is having an active role in control of *B. subtilis* in this species. Therefore, while this data is interesting, it also gives a clear starting point for future studies where data on other immune components would complement the findings from this experiment.

This assumption of some control of bacterial numbers by the host is supported in Experiment 1b, where we see linear growth of *B. subtilis* in the haemolymph, as opposed to the exponential growth curve expected from rapidly dividing bacteria. Inhibition must be taking place to some extent to prevent total colonisation of the haemocoel. If PO was upregulated, perhaps the bacteria would be combatted by the immune system to a greater extent. Future studies considering phagocytosis/nodulation and potential AMP upregulation would be beneficial in analysing the costs of balancing immune investment and parasite control. Studies measuring a longer timeframe of *B. subtilis* replication in the haemolymph would also be beneficial in case clearance eventually occurs, or that the timeframe used in this experiment is still within that of the lag phase for *B. subtilis* growth.

Furthermore, we saw in the survival study on virgin females treated with *B. subtilis* that there was no survival cost to harbouring the parasite. *B. subtilis* seems to have very low virulence in the interaction with *N. vespilloides*. Therefore, the costs of employing the immune response might not outweigh the benefits of bacterial clearance. We know that personal immune function in this species is costly, as it trades-off against reproduction [39]. Perhaps the optimum strategy is not to invest in complete bacterial clearance (sterilising immunity) if the bacteria do not have toxic side effects but simply divert resources from the beetle for their replication and maintenance. If the cost of these diverted resources, alongside keeping the bacteria below a threshold level, is less than the cost of employing the immune system to clear them, this strategy will be selected for, *i.e.*, we would expect immune optimisation such that low intensity infections would be tolerated if the costs of sterilising immunity outweigh the benefits [52]. However, being a lab study, there is a lack of additional costs that would be present in the field. Perhaps a survival effect of possible diverted resources and a limited immune response would be found in the field, where it is more difficult for the organism to compensate for a loss in resources and a lack of investment in immunity. *Ad libitum* feeding conditions in the lab may have masked costs. In summary, it seems that the combination of a non-pathogenic bacteria coupled with favourable conditions in the lab may not be challenging enough to reveal a trade-off.

However, one clear observed effect of *B. subtilis* injection was that the different treatment groups resulted in changes in reproductive investment. Across the full range of carcass weights, in contrast to our hypothesis, reproductive output was lower in control (nutrient broth) beetles. Beetles in the nutrient broth-treated group had a lower reproductive output (total brood weight) at high carcass weights, predominantly because they have fewer larvae as carcasses get larger. In contrast, in the bacteria-treated group the number of larvae was maintained with carcass weight, alongside a marginal increase in mean larval weight across all carcass weights. Across a narrow range of carcass weights where the data cluster, total brood weight is greater in the high bacteria-treated group, relative to controls, which is driven by a marginal increase in mean larval weight for the bacteria-treated beetles. Both analyses support the experiment by Cotter *et al.* [34] in which reproductive restraint is lifted by the challenge of dead bacteria, and the total brood weight also increases. The same pattern holds true in this study with live bacteria, albeit a bacterium that is not that virulent. This challenge may provide a signal to the individual that their survival could be at risk and to therefore invest more in the reproductive bout. This seems counterintuitive, as *B. subtilis* does not affect the beetles’ survival. However, it could act as a signal of a detrimental environmental change resulting in a change in the schedule of reproductive investment. Invasion by a high concentration of one bacterium might stimulate a generic reaction to what is likely to be a bacteria-rich environment *i.e.*, either put strategies in place to control higher bacterial numbers or change the balance in life-history traits to optimise fitness in the changing environment, e.g., channel resources into reproduction. We would expect this genus to be tolerant to and/or sensitive to changes in bacterial load and bacterial diversity due to the microbe rich environment it lives and breeds in. For example, they may be adapted to minimising harm from commonly encountered bacteria but may also be sensitive to changing bacterial diversity or load so that strategies can be put in place to respond to challenges or utilise them as environmental cues. This study supports evidence in other taxa for immune challenge resulting in increased reproductive investment. For example, in male mealworm beetles, *Tenebrio molitor*, an immune challenge resulted in an increase in pheromone signalling [53]. This results in increased attractiveness to females following a threat to survival. This response has also been shown in bird taxa; in the blue-footed booby, *Sula nebouxii*, when older males were challenged with lipopolysaccharides, reproductive success increased [54]. Our experiment provides an initial insight into the response of burying beetles to live bacterial challenge, which will be useful in conjunction with future studies considering other live challenges/infection dynamics and different breeding designs.

In the high bacteria treatment group, lytic activity was maintained across all carcass sizes, whereas there appeared to be a decline in lytic activity with carcass size for nutrient broth and low bacteria-treated beetles. This provides one of the potential mechanisms for the heavier broods produced in high bacteria-treated beetles; they are able to produce larger/heavier broods because they are maintaining their lytic activity on larger carcasses, likely resulting in less carcass degradation which produces a more suitable environment for larval growth. Lysozyme is the main component of the exudates that has been shown to have a positive effect on carcass preservation. However, other antimicrobial substances have been identified, which likely contribute to the prevention of decay [55]. In the nutrient broth and low bacteria treatment groups, lytic activity declines across carcass weight in a similar pattern. However, a greater total brood weight is being produced in the low bacteria treatment group, at higher carcass weights, compared to nutrient broth-treated beetles. Although lytic activity is similar in these two groups, another mechanism must be acting to increase the total brood weight produced on large carcasses in low bacteria-treated beetles, such as more eggs being laid or fewer larvae dying/better quality larvae being produced due to investment in other parental care behaviours.

We would expect an increase in parental investment to have costs later on in life. These could manifest themselves as a reduced reproductive output in later broods or a reduced longevity. While lifetime reproductive success (LRS) was not measured, we did focus on survival to determine if increased reproductive investment resulted in a shorter lifespan. There was no effect of this, and as in a similar study looking at the effect of immune elicitors on LRS [39], it is likely that costs were recouped in other areas. For example, this study was carried out in *ad libitum* feeding conditions, which may mask costs and being a lab study, some costly processes that would occur in the field are bypassed, such as flight and carcass location.

While the challenge by *B. subtilis* has provided an interesting insight into the effect non-virulent bacteria can have on life-history traits, future studies firstly identifying and then using different concentrations of more virulent bacteria on *Nicrophorus* would give greater information as to how immunity and reproduction are balanced. This would provide a fuller picture as to when to employ the immune response, e.g., it may be employed for pathogens that would be beneficial to clear (*i.e.*, low concentrations of virulent bacteria), but for high concentrations of virulent bacteria, an immune response may be futile and a complete diversion of resources to reproduction may be observed. While *B. subtilis* was selected due to its ecological relevance, perhaps it was too ecologically relevant and it may have been better to use a more uncommon soil bacterium in retrospect, to unveil trade-offs in reproductive investment and immune investment. Comparative studies, both behavioural and physiological, on related beetles less likely to encounter such high bacterial loads would be interesting to determine if evolution in this particular environment has produced mechanisms by which to deal efficiently with these challenges. We would predict burying beetles to be tolerant and/or resistant to many microbes, but in the evolutionary arms race, microbes have the advantage due to their shorter generation times. There are clearly some microbes virulent to burying beetles and it would be interesting to identify their virulence traits. This might provide insight into how burying beetles combat those that are less virulent, which might have useful consequences for vertebrate, and even human research.
